# HIF-1α Transgenic Bone Marrow Cells Can Promote Tissue Repair in Cases of Corticosteroid-Induced Osteonecrosis of the Femoral Head in Rabbits

**DOI:** 10.1371/journal.pone.0063628

**Published:** 2013-05-13

**Authors:** Hao Ding, You-Shui Gao, Chen Hu, Yang Wang, Chuan-Gui Wang, Ji-Min Yin, Yuan Sun, Chang-Qing Zhang

**Affiliations:** 1 Department of Orthopedic Surgery, Shanghai Sixth People’s Hospital, Shanghai Jiao Tong University, Shanghai, China; 2 Shanghai Key Laboratory of Regulatory Biology, School of life Sciences, East China Normal University, Shanghai, China; Van Andel Institute, United States of America

## Abstract

Although corticosteroid-induced osteonecrosis of the femoral head (ONFH) is common, the treatment for it remains limited and largely ineffective. We examined whether implantation of hypoxia inducible factor-1α (HIF-1α) transgenic bone marrow cells (BMCs) can promote the repair of the necrotic area of corticosteroid-induced ONFH. In this study, we confirmed that HIF-1α gene transfection could enhance mRNA expression of osteogenic genes in BMCs *in vitro*. Alkaline phosphatase activity assay and alizarin red-S staining indicated HIF-1α transgenic BMCs had enhanced osteogenic differentiation capacity *in vitro*. Furthermore, enzyme linked immunosorbent assay (ELISA) for VEGF revealed HIF-1α transgenic BMCs secreted more VEGF as compared to normal BMCs. An experimental rabbit model of early-stage corticosteroid-induced ONFH was established and used for an evaluation of cytotherapy. Transplantation of HIF-1α transgenic BMCs dramatically improved the bone regeneration of the necrotic area of the femoral head. The number and volume of blood vessel were significantly increased in the necrotic area of the femoral head compared to the control groups. These results support HIF-1α transgenic BMCs have enhanced osteogenic and angiogenic activity *in vitro* and *in vivo*. Transplantation of HIF-1α transgenic BMCs can potentially promote the repair of the necrotic area of corticosteroid-induced ONFH.

## Introduction

Corticosteroid-Induced osteonecrosis of the femoral head (ONFH) is one of the most serious complications following corticosteroid treatment. This specially applies when corticosteroids are administered to treat autoimmune diseases such as systemic lupus erythematosus (SLE), nephrotic syndrome and rheumatoid arthritis. The pathogenic mechanism of ONFH might relate to intravascular thrombus occlusion and extravascular marrow lipid deposition, which results in an impaired function of blood supply system. The impaired blood supply system induces local ischemia, osteocyte death and eventually results in subchondral bone collapse [1], [2]. Once the subchondral bone of the femoral head collapses, the only effective treatment is hip arthroplasty, which had poor results for prosthetic durability in young patients with ONFH [3]. Therefore, an early treatment prior to the collapse is a better choice for the disease. Protected weight-bearing, core decompression, rotational osteotomy and vascularized bone grafting have been employed to treat early-stage ONFH, however, the clinical outcomes are not satisfactory [4], [5].

Mesenchymal stem cells (MSCs) are characterized as undifferentiated cells and possess a mesodermal differentiation potential. They are an attractive cell source for the regeneration of damaged tissues in clinical applications and have been used in the treatment of myocardial infarction, nerve injury, bone defect and other diseases [6]–[8]. Previous studies found implantation of MSCs was able to augment neovascularization and bone regeneration in the necrotic area of ONFH [9]–[11]. The possible explanation for the therapeutic effect of MSCs transplantation is that transplanted MSCs can differentiate into osteoblasts to promote bone regeneration directly *in vivo* [12]. The other explanation is MSCs could secret many angiogenic growth factors, which can promote local angiogenesis [13], [14]. Angiogenesis is a fundamental step for bone formation and affluent neovascularization is beneficial to the new bone regeneration. However, the effect of MSCs transplantation is still not fully satisfactory, especially when the necrosis of the femoral head is extensive.

Hypoxia inducible factor-1α (HIF-1α) is a crucial mediator of the adaptive cell response to hypoxia. It plays an important role in angiogenesis-osteogenesis coupling during bone regeneration [15]. Previous study reported that HIF-1α transfection enhanced the capability of BMCs to promote osteogenesis and angiogenesis *in vitro* [16], [17]. Therefore, transplantation of HIF-1α transgenic BMCs appears to be a better approach to treat ONFH. In this study, we tested the hypothesis that transplantation of HIF-1α transgenic BMCs into the necrotic area of the femoral head could potentially promote the repair of the necrotic tissues in a rabbit model with early-stage corticosteroid-induced ONFH.

## Materials and Methods

### Rabbits

Male New Zealand white rabbits ranging in an age from 8 to 16 weeks and weighing 2–3 kg were used. The rabbits were kept in single cages with controlled temperature (20–22°C) on a 12 h light/dark cycle and were fed a standard diet. The study protocol adhered to the recommendations of the US Department of Health for the care and use of laboratory animals and was approved by the Ethics Committee of Shanghai Jiao Tong University.

### Isolation and Culture of BMCs

Primary rabbit BMCs were harvested by flushing the bone marrow cavity of the femurs and tibias of the rabbits with Dulbecco’s modified Eagle’s medium (DMEM) (GIBCO BRL, Grand Island, NY, USA). The cell suspension was plated and cultured in DMEM supplemented with 10% FBS and 100 U/ml penicillin/streptomycin at 37°C in a humidified 5% CO_2_ incubator. The culture medium was replaced every 3–4 days with non-adherent cells removed. The cells were trypsinized and passaged approximately at a 1∶3 split at subconfluence. The cells from the third passage were used for transduction with lentivirus and following experiments.

### Construction of the Lentiviral Vector

Lenti-HIF-1α and Lenti-GFP vectors were constructed. The former vector encoded green fluorescent protein (GFP) and a specific form of HIF-1α. However, the latter vector only encoded GFP. Total RNA was extracted according to the protocol provided by the PrimeScript RT reagent Kit (TAKARA-BIO, Shiga, Japan). Complementary DNA (cDNA) was synthesized using Reverse Transcription System Kit (Invitrogen, Carlsbad, CA, USA). Primers for polymerase chain reaction (PCR) were designed using Primer Premier 5.0 software and based on HIF-1α mRNA sequences from GenBank (NM_001530.3). The full-length HIF-1α gene was isolated by PCR and inserted between the BamHl and Xbal sites of the viral vector pLVX-IRES-ZsGreen1 (Clontech, CA, USA). The vector was cotransfected with PMD2.G and PSPAX2 into 293T cells for lentivirus production. The viruses were collected on Day 2 and 3 after the transfection and were concentrated to up to 100 folds by ultracentrifugation.

### Gene Transduction

BMCs were plated in 10-cm dishes at a density of 1×10^6^ cells in 5 ml medium and transfected with the lentivirus (Lenti-HIF-1α and Lenti-GFP) at MOI (multiplicity of infection) of 50 PFU/cell in the presence of 6 µg/ml polybrene (Sigma Aldrich, St Louis, MO, USA). The medium was replaced with fresh medium 24 hours after transfection. Transfection efficiency was analyzed by calculating number of green cells among all cells on Day 3 after transfection. To achieve more accurate results, GFP-positive cells were sorted out by FACS and these sorted cells were used in the following *in vitro* and *in vivo* experiments. Then, overexpression of HIF-1α in these cells was confirmed using western blotting.

### Quantitative RT-PCR Analysis

Quantitative RT-PCR was carried out to detect the expression of related genes in Lenti-HIF-1α-transduced BMCs at 4, 7, 14, 21 and 28 days after transduction. Total RNA was extracted using the Trizol method (Invitrogen, Carlsbad, CA, USA). One microgram of pure RNA, as assessed spectrophotometrically using the A260/A280 ratio, was reverse transcribed using PrimeScript RT reagent kit. Two microliters of the reverse transcription reaction were mixed with iQ SYBR Green super mix and amplified using iQ5 real-time system. The product was quantified using a standard curve and all values were normalized to β-actin. Lenti-GFP-transduced BMCs and normal BMCs were analyzed as control. Primer pairs for amplification are listed in Table 1. All reactions were performed in triplicate.

**Table 1:** Quantitative real-time polymerase chain reaction primer sequences.

Gene	Forward primer	Reverse primer
HIF-1a	GTTCACCTGAGCCTAATAGTCC	GGAACGTAACTGGAGGTCATC
VEGF	GGCAGAAGAAGGAGACAATAAACC	CAGAGGCACGCAGGAAGG
OCN	TGACAAAGCCTTCATGTCCA	TGCCAGAGTTTGGCTTTAGG
ALP	ATGGGATGGGTGTCTCCACA	CCACGAAGGGGAACTTGTC
B-actin	CCATCTACGAGGGCTACGC	CGGCTGTGGTCACGAAGG

### ELISA for VEGF Production

*In vitro* VEGF production by Lenti-HIF-1α-transduced BMCs during a 24-h period was quantified in culture medium at 4, 7, 14, 21 and 28 days after transduction using ELISA kit (antibodies-online, Aachen, Germany), according to the manufacturer’s instructions. Briefly, the cells were trypsinized and split into 6-well plates at 3×10^5^ cells/well. The medium was changed with fresh medium on the following day. After incubation for 24 h, the medium was harvested for ELISA. Total protein content of the cells was determined for standardization of VEGF production with BCA protein assay kit (Pierce Biotechnology, Rockford, IL). All experiments were performed in triplicates.

### Alkaline Phosphatase (ALP) Activity and Alizarin Red-S Staining

*In vitro* osteogenic differentiation capacity was confirmed by the detection of ALP activity and the deposition of mineralized matrix. Cells were osteogenically induced using StemPro Osteogenesis Differentiation Kit (GIBCO BRL, Grand Island, NY). ALP activity was determined by biochemical colorimetric assay using alkaline phosphatase kit (Gene Tex, Irvine, CA, USA) 14 days after osteogenic induction, according to the manufacturer’s instructions. Values were normalized against protein concentration, which was measured with a BCA protein assay kit (Pierce Biotechnology, Rockford, IL, USA). Alizarin red-S staining was performed 21 days after osteogenic induction. Cell cultures were washed twice in PBS and were fixed in 70% ethanol for 30 minutes. The cultures were then stained with alizarin red-S for 30 minutes and were evaluated by light microscopy. Calcium deposition was confirmed by the formation of red nodules. Quantification was performed with a colorimetric assay. The cultures were incubated with 20% methanol/10% acetic acid solution for 15 minutes followed by optical density determination at 450 nm. Values were normalized against protein concentration. All experiments were performed in triplicates.

### Rabbit ONFH Model and Treatment Protocol

Rabbit ONFH model was established according to the reported protocols [18]. In brief, rabbits were injected with 10 µg/kg of lipopolysaccharide (LPS; Sigma) intravenously. They were then given three injections of 20 mg/kg of methylprednisolone (MPS; Pfizer, USA) intramuscularly at a time interval of 24 h. Osteonecrosis of the femoral head gradually developed and was confirmed with magnetic resonance imaging six weeks after injection of MPS, which was categorized as stage II based on Steinberg criteria. The Stage of ONFH was evaluated by two experienced radiologists in a blinded fashion respectively. Then the rabbits were randomly divided into four groups. Group I (n = 9) underwent bilateral core decompression of the femoral head and Lenti-HIF-1α-transduced BMCs transplantation. Group II (n = 9) underwent bilateral core decompression and normal BMCs transplantation. Group III (n = 9) underwent bilateral core decompression. Group IV (n = 9) did not receive any therapy.

The surgical procedure was performed as described previously [9]. Briefly, a standard lateral approach was made to expose the lateral aspect of the femur distal to the greater trochanter. A drill with an outer diameter of 1 mm was inserted at the flare of the greater trochanter and into the necrotic area of the femoral head. The necrotic tissue was then removed. The location was confirmed using a C-arm x-ray machine. For cell transplantation, a total of 5×10^6^ cells were resuspended in 0.5 ml PBS. This was injected into the femoral head through the bone tunnel made by drill. The tunnel was sealed by an absorbable collagen sponge plug. The wound was then closed layer by layer. After the surgery, animals were placed in recovery cages and allowed to bear weight immediately.

### Perfusion and Decalcification

Four weeks after surgery, the samples were perfused and decalcified according to the previous protocols [9]. In brief, the animals were anesthetized with 3% sodium pentobarbital (1 ml/kg) and the abdomen cavity was opened. Microfil (Microfil MV-122, Flow Tech; Carver, MA) was injected in the vasculature of the femoral head using a scurg needle from the abdominal aorta. The rabbits were then euthanized and stored at 4°C for 1 h to ensure polymerization of the contrast agent before microangiography.

### MicroCT Scanning

The Proximal parts of the femurs were harvested and fixed in paraformaldehyde (4%). Then they were scanned by microCT with a high-resolution system to evaluate bone healing. Briefly, the samples were scanned continuously at a resolution of 25 µm per voxel. The region of interest (ROI) was selected with the aid of preoperative MRI. The images were segmented using a low pass filter to remove noise and bone tissue was defined at a threshold of 800. The parameters of bone volume (BV), tissue volume (TV) and bone mineral density (BMD) of ROI were then calculated. The ratio of BV to TV indicates the relative amount of bone of the selected ROI. BMD represented the mean bone density of the selected ROI.

These samples were then decalcified with ethylene diamine tetra acetic acid (EDTA, 10%, PH 7.4) and scanned again to measure the vascularization of the femoral head. The scan was performed at a resolution of 36 µm per voxel with 1024×1024 pixel image matrix. Noise was removed with a low pass Gaussian filter and blood vessels were then defined at a threshold of 85. The blood vessels filled with Microfil were included with semi-automatically drawn contour at each two-dimensional section by built-in “Contouring Program” for automatic reconstruction of 3-D image of vasculature in the sample. In addition, axial slices through the samples were sequentially visualized and the number of vessels penetrating the bone vessels was calculated. All voxels counted in the specified microfil range represented the vessel volume.

### Histology, Immunohistochemistry and Immunofluorescence

After microCT, the samples were embedded in paraffin and sectioned at 5 µm. The sections from each group were stained with hematoxylin-eosin and analyzed with a light microscope. New bone formation was measured with image-analysis software (NIH image) and its density was defined as the ratio of new bone area to total implant area ×100. New bone calculations did not include osteoid tissue since the border of the new bone and osteoid tissue was unclear and the inclusion of osteoid tissue could lead to overestimation.

Immunohistochemistry for CD31 (ab9498, abcam, 1∶200) was also performed to measure new blood vessels in the necrotic area of each group. Briefly, the sections were rehydrated, treated with antigen retrieval and incubated with the primary antibody at 4°C overnight. Subsequently, the biotinylated secondary antibody and ABC complex were applied and DAB substrate was used to stain the sections. The sections were then treated with hematoxylin and mounted. Vessels were defined by positive CD31 stain and their typical round or oval structure.

To confirm the implantation of the transfected BMCs in the necrotic area and their survival, sections were stained with DAPI (4,6-diamidino-2-phenylindole, Vector Laboratories, Burlingame, CA) and were analyzed with fluorescence microscope (Leica, Bannockburn, IL). To confirm *in vivo* differentiation of the transfected BMCs into osteoblasts, immunofluorescence was performed in sections from Group I for OCN (ab13420, abcam, 1∶100). Briefly, the sections were treated with antigen retrieval solution and were blocked with 5% BSA-PBS for 30 min at room temperature. They were then incubated with the primary antibody at 4°C overnight, followed by incubation with Alexa Fluor 594 goat anti-mouse secondary antibody (Invitrogen, 1∶400) for 1 h. Finally, the Vectashield Mounting media was applied to the sections and they were examined with fluorescence microscope.

### Statistical Analysis

Data were presented as mean ± standard error. Statistical analysis of the data was performed using ANOVA with a SNK post-hoc analysis. *P*-value <0.05 was considered statistically significant.

## Results

### Gene Transduction and HIF-1α Overexpression

**Figure 1:**
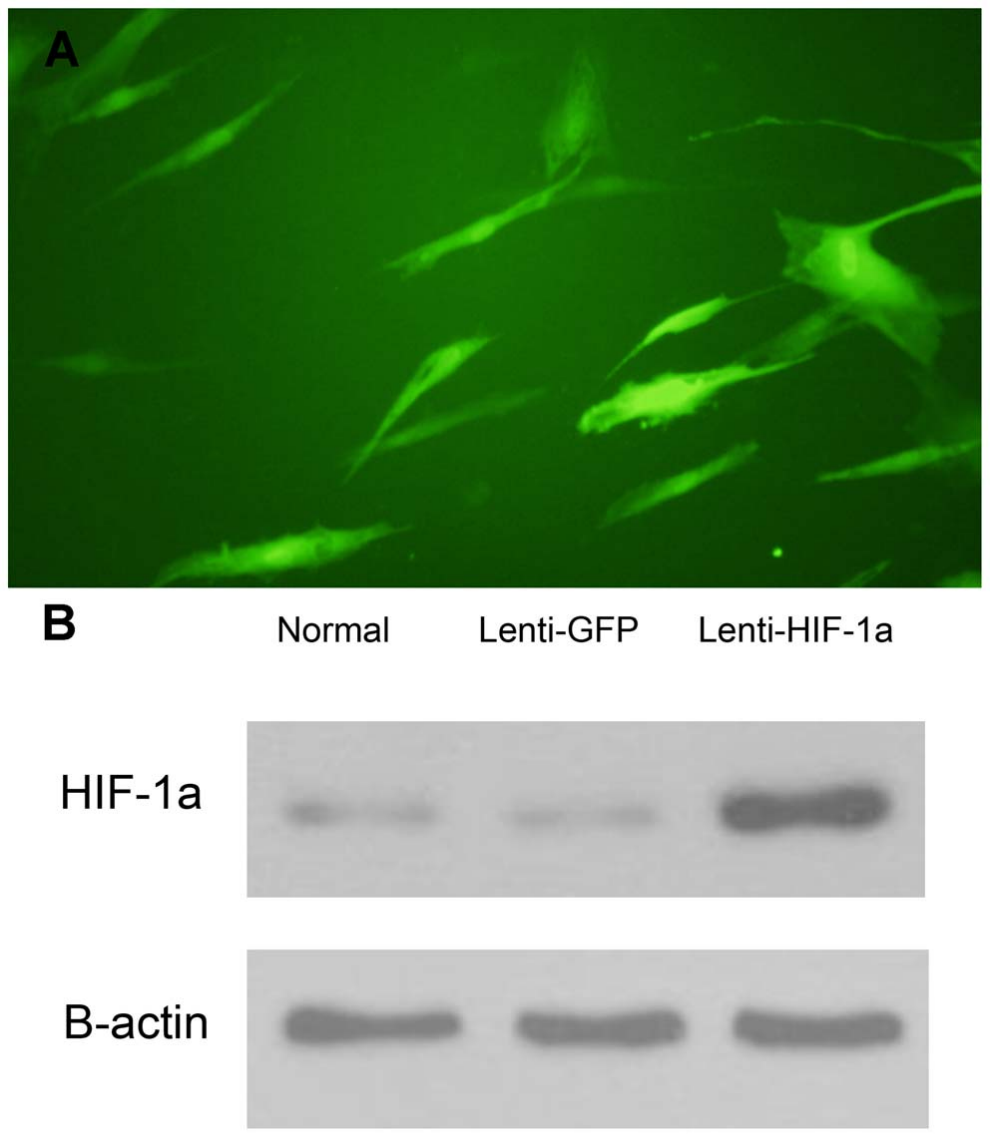
The transduced BMCs and the western blotting to dectect the expression of HIF-1a. (A): The transduced BMCs were sorted with FACS and checked by fluorescence microscope. (B): The western blotting confirmed the overexpression of HIF-1a in Lenti-HIF-1a-transduced BMCs and there was nearly no expression in Lenti-GFP-transduced BMCs and normal BMCs.

Through preliminary experiments, we found MOI of 50 resulted in optimal infection efficiency without excessive cell death. Three days after transduction, about 80% of the cells were green when observed under fluorescence microscopy. To get more accurate results, we used FACS to sort out virus-infected positive cell with GFP. The single cells were checked by fluorescence microscope with about 100% infection (Figure 1A). The western blotting confirmed the overexpression of HIF-1α in Lenti-HIF-1α-transduced BMCs (Figure 1B).

### HIF-1a Expression Induces Related Gene Expression *In Vitro*

**Figure 2:**
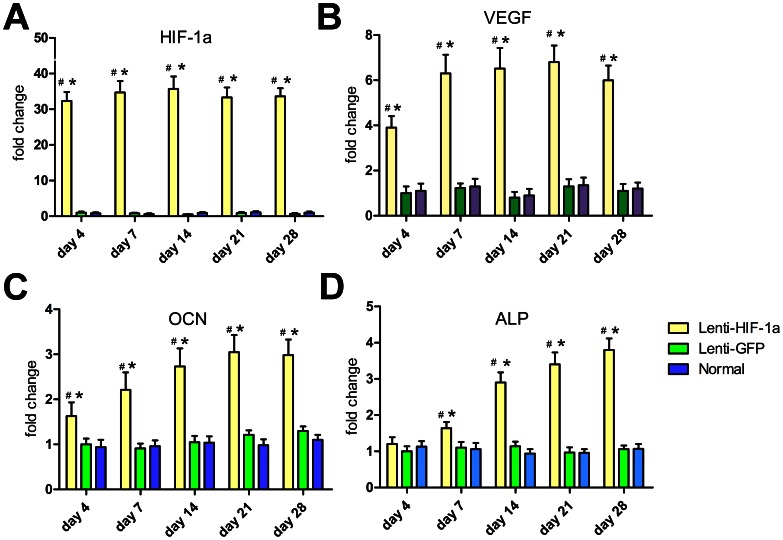
Gene expression of related factors in transduced and normal BMCs. The mRNA expression level of HIF-1a (A), VEGF (B), OCN (C) and ALP (D) at day 4, 7, 14, 21 and 28 after transduction. #, p<0.01 (compared with Lenti-GFP-transduced BMCs) and *, p<0.01 (compared with normal BMCs). Abbreviations: HIF-1a, hypoxia-inducible factor-1 alpha; VEGF, vascular endothelial growth factor; OCN, osteocalcin; ALP, alkaline phosphatase.

Expression of related genes in Lenti-HIF-1α-transduced BMCs was detected by quantitative RT-PCR on Day 4, 7, 14, 21 and 28 (Figure 2). Transcription level of these genes showed notable difference among various groups. Expression of HIF-1α gene in Lenti-HIF-1α-transduced BMCs maintained a high level during the four weeks, while there was nearly no expression in Lenti-GFP-transduced BMCs and normal BMCs. Expression of VEGF gene in Lenti-HIF-1α-transduced BMCs had a 3-fold increase compared to the other two groups on Day 4 and a 5-fold increase during the last three weeks. OCN was increased 60% as compared to the other two groups on Day 4 and had another gradual increase of two folds from Day 4 to 21. The ALP transcription level in Lenti-HIF-1α-transduced BMCs had a steadily increase from Day 4 to Day 28, which maintained a low level in other two groups.

### HIF-1a Expression Induces VEGF Expression *In Vitro*

**Figure 3:**
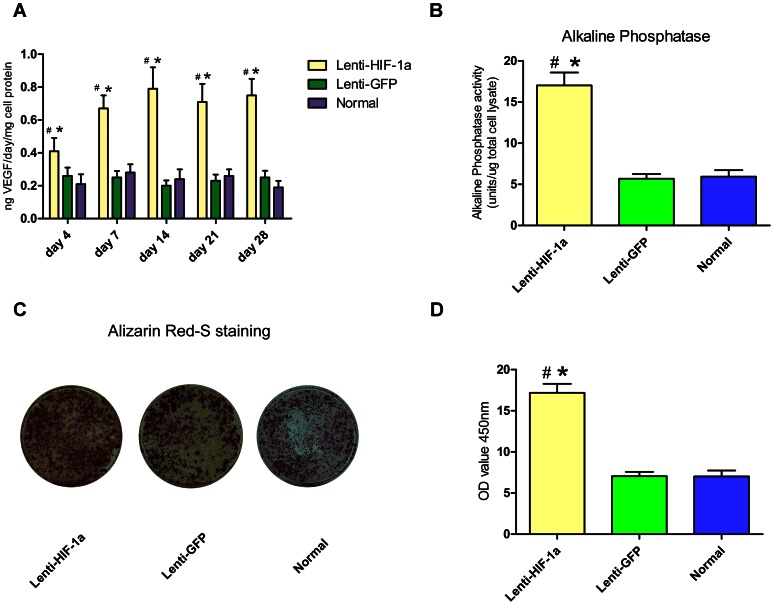
VEGF production, Alkaline Phosphatase assay and Alizarin red-s staining *in vitro*. (A): VEGF production in Lenti-HIF-1a-transduced BMCs was significantly increased as compared to that in Lenti-GFP-transduced BMCs and normal BMCs for at least 28 days. (B): Alkaline Phosphatase activity in Lenti-HIF-1a transfected BMCs was about 2-fold higher than that in Lenti-GFP transduced BMCs and normal BMCs 14 days after osteogenic induction. (C): Alizarin red-S staining showed more calcium deposits in Lenti-HIF-1a-transduced BMCs 21 days after osteogenic induction. (D): Quantitative analysis revealed an increase of about 1.5-fold in extracellular matrix mineralization in Lenti-HIF-1a-transduced BMCs compared to Lenti-GFP-transduced BMCs and normal BMCs. #, p<0.01 (compared with Lenti-GFP BMCs) and *, p<0.01 (compared with normal BMCs).

The level of VEGF that was released into the culture medium from BMCs was directly measured by ELISA method on Days 4, 7, 14, 21 and 28 after transduction. Figure 3A represents that VEGF production of Lenti-HIF-1α-transduced BMCs was significantly increased on Day 4 compared to that of Lenti-GFP-transduced BMCs and normal BMCs (p<0.01). It gradually increased and was maintained at 4 folds during the last three weeks. The results were in line with the findings in quantitative RT-PCR analysis.

### HIF-1a Expression Promote Osteogenesis *In Vitro*

ALP assay demonstrated that the activity in Lenti-HIF-1α-transduced BMCs was about two folds higher than that in Lenti-GFP-transduced BMCs and normal BMCs (Figure 3B). Alizarin red-S staining was employed to detect mineralization of extracellular matrix at later time point. Massive calcium deposits in Lenti-HIF-1α-transduced BMCs appeared on Day 21, while just a small amount of calcium deposits were present in Lenti-GFP-transduced BMCs and normal BMCs (Figure 3C). After spectromorphometric quantification and protein normalization, Lenti-GFP-transduced BMCs showed an increase of about 1.5 folds in extracellular matrix mineralization as compared to the Lenti-GFP-transduced BMCs and normal BMCs (Figure 3D).

### HIF-1a Expression Promote Bone Regeneration *In Vivo*

**Figure 4:**
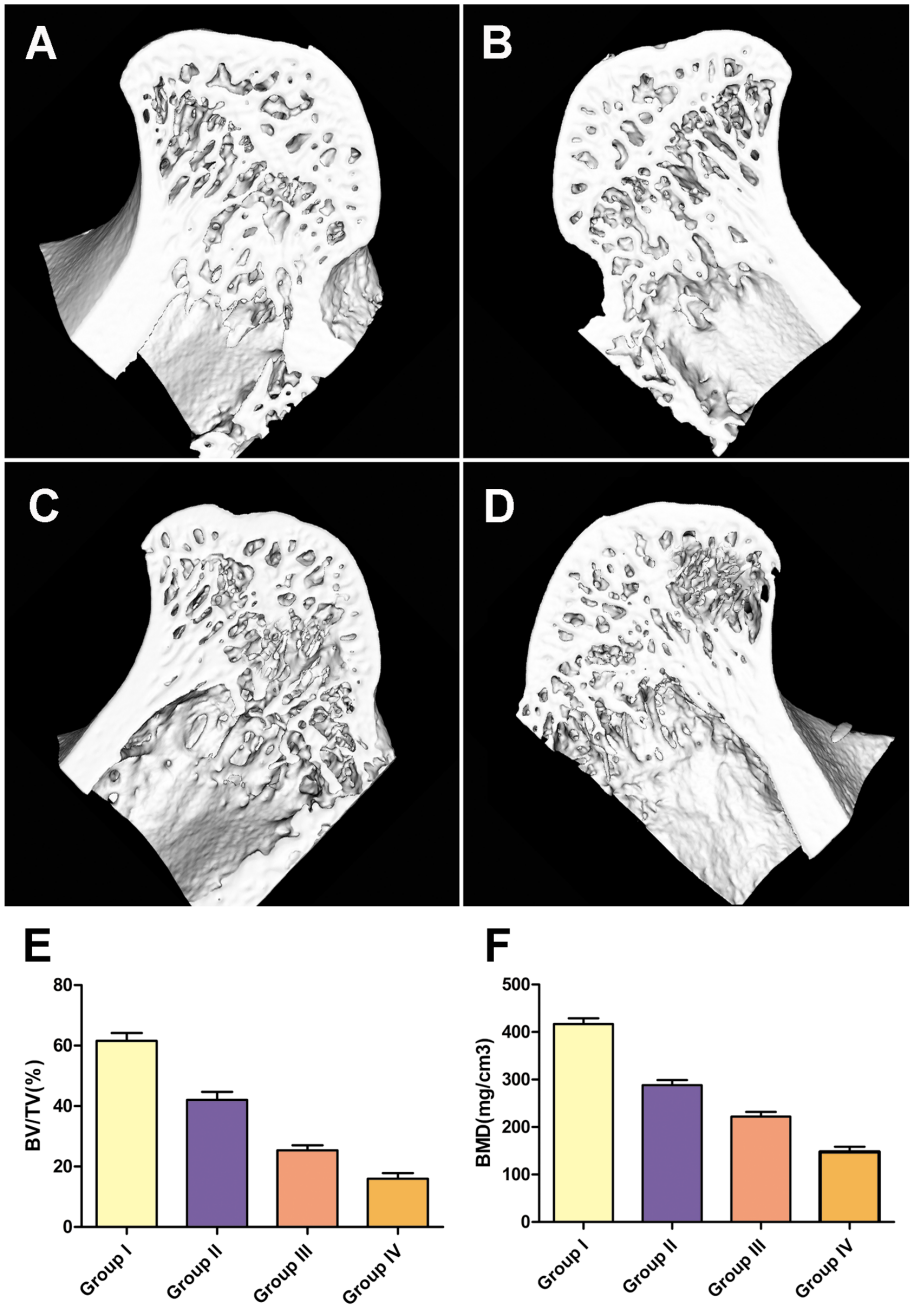
Representative three-dimensional images of the femoral head obtained by microCT. The subchondral trabeculae of the femoral head appeared intact and well distributed In Group I and II (A, B). The subchondral trabeculae became thin and sparse In Group III (C). In Group IV, local destroyed trabeculae could be observed with a fewer amount (D). The quantitative analysis of microCT showed significantly higher values in Group I than other three groups in BV/TV and BMD (p<0.01) (E and F). BV, bone volume; TV, trabecular volume; BMD, bone mineral density.

Bone healing was firstly evaluated with micro-CT. In Group I and II, which received the transplantation of Lenti-HIF-1a-transduced BMCs and normal BMCs respectively, the subchondral trabeculae of the femoral head appeared intact and well distributed. In Group III, which only received core decompression, the subchondral trabeculae became thin and sparse. In Group IV, which did not received any treatment, local destroyed trabeculae could be observed with a fewer amount. The date of quantitative analysis showed significantly higher values in Group I than other three groups in both BV/TV and BMD (Figure 4).

**Figure 5:**
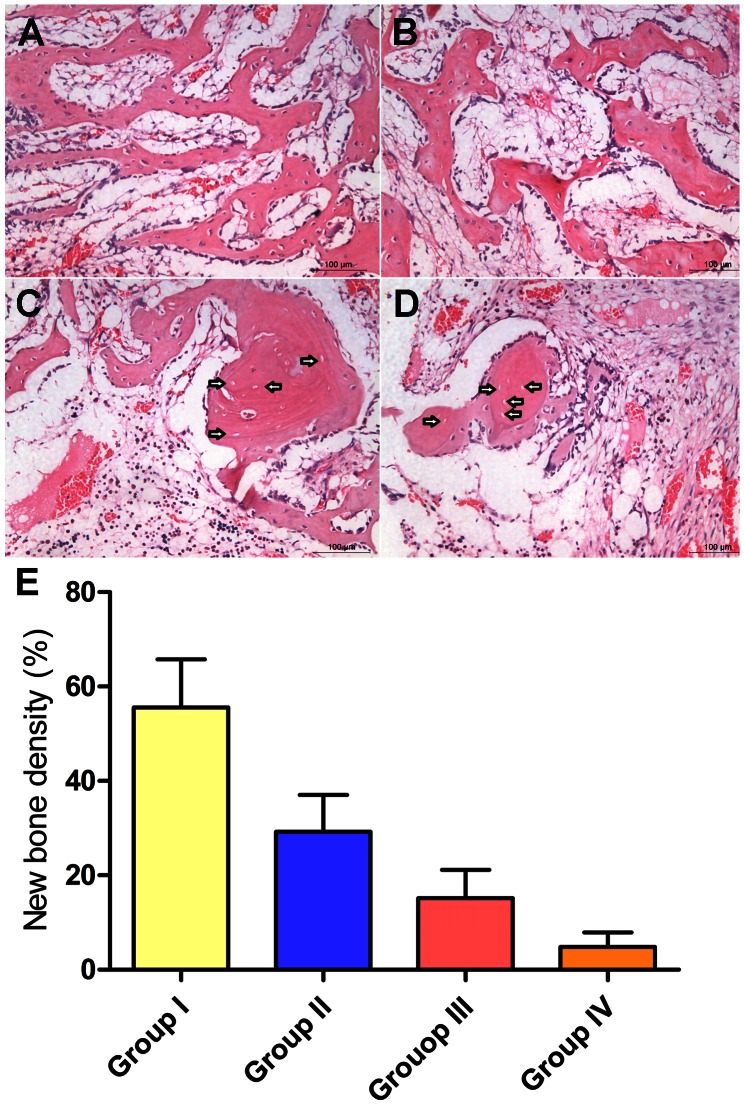
Assessment of new bone regeneration in the necrotic area. Representative histological photomicrograph of the necrotic area of the femoral head showed massive trabecular tissue had formed in Group I and there were few granulation tissue (A). In Group II, there were fewer trabecular tissue and more granulation tissue as compared to that in Group I (B). In Group III, a few osteocyte filled lacunae were replaced by empty lacunae. The marrow fat cells were increased compared to that of Group I and II (C). In Group IV, there were rarely any trabecular tissue, and a lot of empty lacunae were observed (D). Scale bars = 50 um. Arrowheads indicate empty lacunae. (E) Histomorphometric analysis showed new bone density in Group I was significantly more than that of the other three groups (p<0.01).

The H&E staining showed that new bone and massive trabecular tissue had been induced in the samples of Group I. A large number of osteocytes were distributed along the trabeculae. Furthermore, there was few granulation tissue (Figure 5A). In Group II, there was some disordered trabecular tissue in the necrotic zone and more granulation tissue as compared to that in Group I (Figure 5B). In Group III, a few osteocyte filled lacunae was replaced by empty lacunae. The granulation tissue was increased as compared to that of Group II (Figure 5C). In Group IV, massive fat cells and granulation tissue were observed in the medulla. There was rare trabecular tissue and a lot of empty lacunae that were distributed along the trabeculae (Figure 5D). Histomorphometric analysis of all the conditions confirmed that the bone regeneration in Group I was more than that of the other three groups, which supported the qualitative finding based on histological observations (Figure 5E).

### HIF-1a Expression Promote Neovascularization *In Vivo*

**Figure 6:**
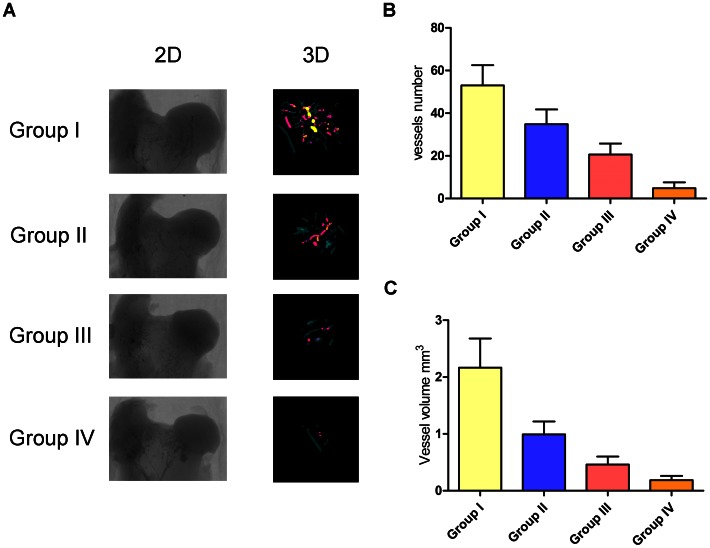
Assessment of femoral head neovascularization. (A): Representative images of microCT 2-D sections and reconstructed 3-D microangiography of the femoral head from various groups. Quantitative analysis of MicroCT showed mean number of blood vessels (B) and vessel volume (C) of Group I were significantly higher than that of the other three groups (p<0.01).

**Figure 7:**
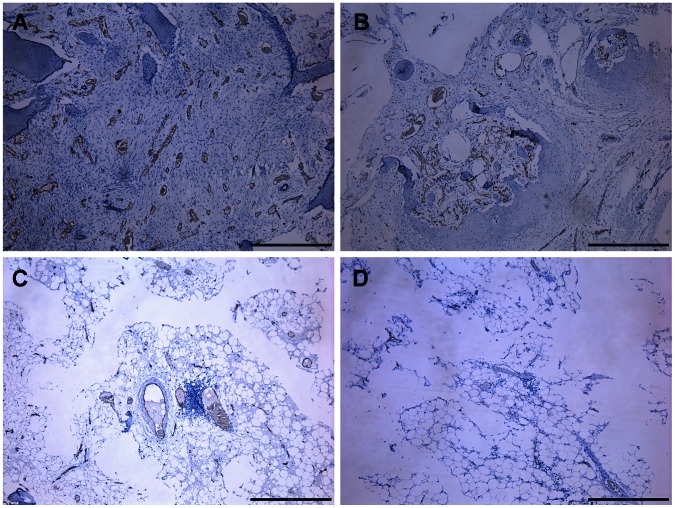
Immunohistochemistry for CD31. There were more CD31-positive vessels observed in Group I (A), compared to that in Group II (B). Few CD31-positive vessels were observed in Group III (C), while nearly none were present in Group IV (D). White arrowheads indicate CD31-positive vessels. Scale bars = 500 um.

Vascular microarchitecture of the femoral head was analyzed using microCT and the results were reconstructed in 3-D for presentation. The samples of Group I had intensive vasculature in and around the femoral head, which was significantly increased as compared to the other three groups. The samples of Group II had local vascular architecture in the femoral head, the density of which was obviously lower than that of Group I. The samples of Group III showed fewer vessels in the femoral head. The samples of Group IV had nearly no sign of vasculature (Figure 6A). The average number of blood vessels and the volume of the vessels penetrating into the necrotic area of the femoral head were quantitatively analyzed. The results are presented in Figure 6B and 6C, which was in line with the above qualitative description. In addition, immunohistochemical experiments revealed numerous CD31-positive vessels were observed in the necrotic area of Group I, which was obviously more than that in the other three Groups (Figure 7).

### Immunofluorescence

**Figure 8:**
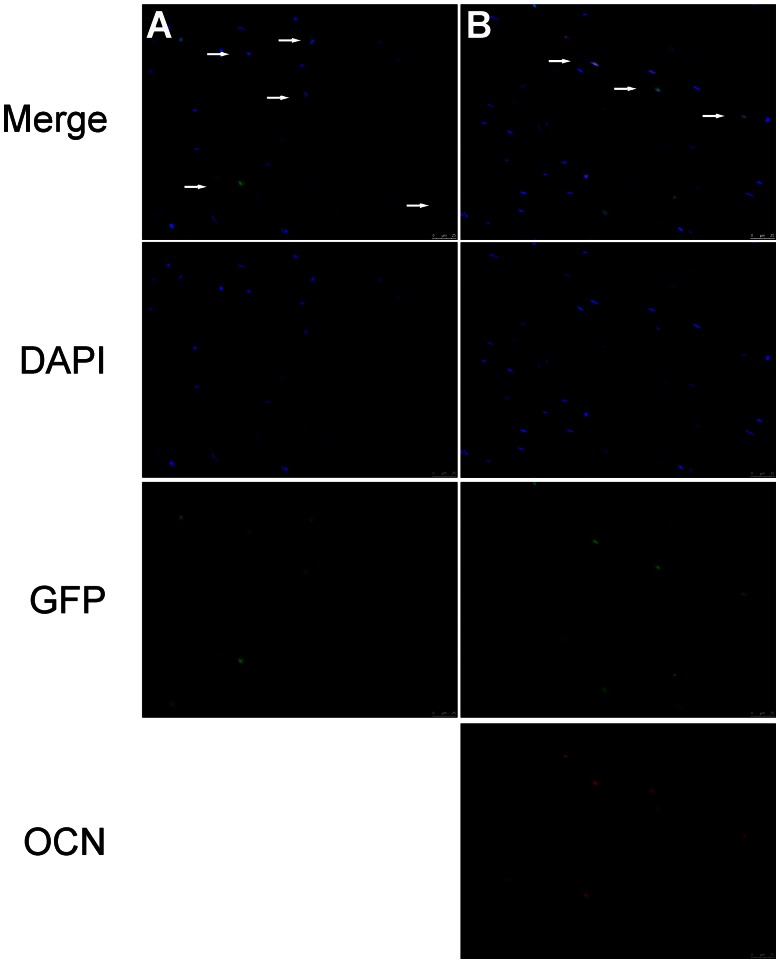
Immunofluorescence of the necrotic area of the femoral head. (A): DAPI and GFP merged pictures revealed the transplanted GFP-positive BMCs existed in the necrotic area of the femoral head in Group I. (B): Immunofluorescence for OCN showed some GFP-positive cells in the necrotic area expressed OCN. White arrowheads indicate colocalization. Scale bars = 25 um. Abbreviations: DAPI, 4,6-diamidino-2-phenylindole; OCN, osteocalcin.

Through immunofluorescence microscopy, massive number of GFP-positive cells was detected in the necrotic area of the femoral heads in Group I, while none were detected in other three groups (Figure 8A). This indicated that the transfected BMCs were implanted in the necrotic area and survived. Also, immunofluorescence studies revealed some GFP-positive cells in the necrotic area of Group I co-stained together with OCN (Figure 8B), which indicated the transplanted BMCs might directly differentiate into osteoblasts *in vivo*.

## Discussion

Corticosteroid-induced ONFH is a debilitating disease in orthopedics, which refers to death of bone cells due to ischemia and hypoxia. Current therapy options for early-stage ONFH include protected weight-bearing, core decompression, rotational osteotomy and vascularized bone grafting. However, none of these can yield satisfactory results.

Some groups of researchers transplanted MSCs into the necrotic area of the femoral head to aid the core compression in order to treat ONFH. Gangji *et al*. followed up 19 patients (24 hips) for five years and found BMCs transplantation could obviously release pain and improve the function of hips in patients with early stages of ONFH [19]. We had used BMCs to treat corticosteroid-induced ONFH in rabbit model with good result [9]. However, there are only a small number of autologous MSCs that could be used to treat ONFH, and a limited number of MSCs that could be amplified *in vitro* to enhance the therapeutic efficacy. Therefore, MSCs with enhanced osteogenic or angiogenic activity are needed to yield a better therapy result.

Gene modification is an important approach of gene therapy. Researchers have reported that the transduction of specific genes into MSCs could dramatically improve their potential of therapeutic efficiency. In a number of studies, bone morphogenetic protein (BMP), VEGF and hepatocyte growth factor (HGF) had been transduced into MSCs to enhance their osteogenic and angiogenic capacity. A certain extent of beneficial effect was reported for these modified cells upon being transplanted into the necrotic area of the femoral head to treat ONFH [20]–[22]. However, angiogenesis and osteogenesis are two tightly coupled processes for bone regeneration during the repair process of ONFH [15]. Previous gene transduction can only enhance the cell osteogenic or angiogenic capacity, separately.

Hypoxia inducible factors (HIFs) belong to the Per/Anrt/Sim subfamily of basic helix-loop-helix transcription factors and are crucial mediators of the adaptive cell response to hypoxia. The HIF family comprises of three functional subunits including: HIF-1α, HIF-2α and HIF-3α, which form a heterodimer with the HIF-1β subunit. HIF-1α arose early in evolution and is widely expressed in most human tissues. It controls the activity of a broad array of genes and modulates stem cell proliferation, differentiation and pluripotency [23]. It is also believed that HIF-1α plays an important role in bone development and regeneration [24], [25].

In previous study, HIF-1α could promote the differentiation of MSCs into osteogenic lineages and enhance their osteogenic activity [16], [26]. HIF-1α could also promote the secretion of VEGF in BMCs. VEGF is a potent mitogen for endothelial cells and plays a central role in angiogenesis and neovascularization [27]. Therefore, transduction of HIF-1α gene into BMCs can enhance osteogenic and angiogenic activity of the cells simultaneously and has more advantages in accelerating bone regeneration as compared to a single angiogenic gene. Based on these results, we hypothesized that HIF-1α-transgenic BMCs had a better effect to promote the repair of the necrotic area in early-stage ONFH.

In this study, HIF-1α gene was transduced into BMCs using lentivirus vectors in order to stably overexpress HIF-1α in the cells. Quantitative RT-PCR and Western blotting were performed to confirm that HIF-1α mRNA and protein were overexpressed in HIF-1α-transgenic BMCs. Quantitative PT-PCR further indicated that HIF-1α transfection could induce BMCs to express osteogenic genes as well as VEGF for at least four weeks. Alkaline phosphatase activity assay and Alizarin red staining revealed that the capacity of HIF-1α-transgenic BMCs for osteogenic differentiation was significantly enhanced as compared to that of normal BMCs. ELISA assays to detect VEGF showed that HIF-1α transfection could largely enhance VEGF production in BMCs for at least four weeks after transfection. These data confirmed that HIF-1α-transgenic BMCs had a better osteogenic and angiogenic capacity *in vitro*.

To detect the capacity of HIF-1α-transgenic BMCs to promote bone tissue regeneration *in vivo*, an experimental rabbit model of early-stage corticosteroid-induced ONFH was established. HIF-1α-transgenic BMCs were transplanted in the necrotic area of the femoral head in rabbits. Through immunofluorescence microscopy, we confirmed that the implanted GFP-positive BMCs could survive in the necrotic area for at least four weeks after the transplantation. Through microCT and immunohistochemistry assays in CD31, we found that there was a significant increase in new blood vessel volume and number in group I as compared to the other three groups. This indicated that HIF-1α-transgenic BMCs had enhanced angiogenic capacity *in vivo*. This might be because HIF-1α-transgenic BMCs could secrete more VEGF. VEGF can induce endothelial cell growth and inhibit endothelial cell apoptosis. It also can promote endothelial progenitor cell migration and angiogenesis [28]. Furthermore, some researchers reported transplanted MSCs to be able to directly differentiate into endothelial cells in order to promote local vascular formation [6], [12].

Angiogenesis is a complex process, which needs the involvement of multiple angiogenic factors. Many researchers believed that the use of a single factor was insufficient to form functional vascular structures [29]. Besides, other than VEGF, HIF-1α could also increase the expression of some other angiogenic factors, such as bFGF, Ang-1, SDF-1, PLGF and SCF [30]–[32]. All of these factors take part in the process of angiogenesis [33]–[35] and the cooperation of these factors would have a better result in promoting new vascular formation in the necrotic area of the femoral head.

The results of microCT and histomorphometric analysis indicated that there was more bone in the necrotic area of Group I, compared to the other three groups. Microscopic examination also revealed that the quality of new bone in group I was better than the other three groups. This might be because HIF-1α transfection promoted osteogenic differentiation capacity of BMCs. Immunofluorescence assays in OCN showed that some GFP-positive cells in the necrotic area could express OCN, which indicated the transplanted BMCs could differentiate into osteoblasts *in vivo*. There were studies indicating that VEGF could promote osteoblast proliferation/differentiation and stimulate chemotactic migration of osteoblasts, which might also contribute to the new bone formation [36]–[38]. The other explanation could be that increased angiogenesis promotes new bone regeneration. There is a close association between angiogenesis and osteogenesis and neovascularization is an important element in bone regeneration. It has been demonstrated that HIF-1α overexpression in BMCs through gene therapy can increase angiogenesis in new bone formation, therefore promote the healing of the bone defect [39].

Although the result of this study was promising, the risk of HIF-1a transgenic, such as tumorigenesis, should be seriously considered before clinic application in the future. In our study, there was no evidence that HIF-1a modified BMCs developed to tumor *in vitro* or *in vivo*. However, the safety of HIF-1a transgene needs to be further evaluated by more studies. The prolyl hydroxylase domain (PHD) inhibitor dimethyloxalylglycine (DMOG), which can decrease PHD hydroxylase activity and stabilize HIF-1a under normoxia, may be an alternative strategy that could be safely used in clinic. In previous studies, PHD inhibitors have been successfully used in some disease models to promote tissues regeneration, including diabetic ulcers and skin flap necrosis [40]–[42]. The effect of DMOG in the osteonecrosis of the femoral head are deserved to be explored in the future.

### Conclusions

In summary, this study demonstrates that HIF-1α transgenic BMCs possess better capabilities of osteogenesis and angiogenesis *in vitro,* and can increase vascularization and bone regeneration in the early-stage osteonecrosis of femoral head in the rabbit model. Therefore, local implantation of HIF-1α transgenic BMCs might be a potentially novel and effective therapeutic option for early-stage corticosteroid-induced ONFH.
